# Urinary sodium concentration predicts time to major adverse coronary events and all-cause mortality in men with heart failure over a 28–33-year period: a prospective cohort study

**DOI:** 10.1186/s12872-022-02830-3

**Published:** 2022-09-02

**Authors:** Anand Ganes, Jessica A. Davis, Jyrki K. Virtanen, Ari Voutilainen, Tomi-Pekka Tuomainen, John J. Atherton, John Amerena, Andrea Driscoll, Dave L. Hare, Gary Wittert, Anu Ruusunen, Wolfgang Marx, Mohammadreza Mohebbi, Adrienne O’Neil

**Affiliations:** 1 Department of General Medicine, University Hospital Geelong, Barwon Health, Bellerine Street, Geelong, VIC 3220 Australia; 2 IMPACT–the Institute for Mental and Physical Health and Clinical Translation, School of Medicine, Deakin University, Barwon Health, Geelong, VIC Australia; 3 Institute of Public Health and Clinical Nutrition, University of Eastern Finland, 70210 Kuopio, Finland; 4 Faculty of Medicine, Royal Brisbane and Women’s Hospital, University of Queensland, Herston, Brisbane, QLD Australia; 5 University Hospital Geelong, Geelong, VIC Australia; 6 Faculty of Health, School of Nursing and Midwifery, Deakin University, Burwood, VIC Australia; 7 Austin Health, Studley Rd, Heidlberg, VIC Australia; 8 Department of Cardiology, Austin Health, Melbourne, VIC Australia; 9 University of Melbourne, Melbourne, VIC Australia; 10 Freemasons Centre for Men’s Health and Wellness, South Australian Health and Medical Research Institute, University of Adelaide, Adelaide, South Australia Australia; 11 Department of Psychiatry, Kuopio University Hospital, Kuopio, Finland; 12 Biostatistics Unit, Faculty of Health, Deakin University, Geelong, VIC Australia

**Keywords:** Heart failure, Biomarker, Prognosis, Translational medical research

## Abstract

**Background:**

Lower urinary sodium concentrations (U_Na_) may be a biomarker for poor prognosis in chronic heart failure (HF). However, no data exist to determine its prognostic association over the long-term. We investigated whether U_Na_ predicted major adverse coronary events (MACE) and all-cause mortality over 28–33 years.

**Methods:**

One hundred and eighty men with chronic HF from the Kuopio Ischaemic Heart Disease Risk Factor Study (KIHD) were included. Baseline data was collected between 1984 and 1989. MACE and all-cause outcomes were obtained using hospital linkage data (1984–2017) with a follow-up of 28–33 years. Cox proportional hazards models were generated using 24-h U_Na_ tertiles at baseline (1 ≤ 173 mmol/day; 2 = 173-229 mmol/day; 3 = 230-491 mmol/day) as a predictor of time-to-MACE outcomes, adjusted for relevant covariates.

**Results:**

Overall, 63% and 83% of participants (n = 114 and n = 150) had a MACE event (median 10 years) and all-cause mortality event (median 19 years), respectively. On multivariable Cox Model, relative to the lowest U_Na_ tertile, no significant difference was noted in MACE outcome for individuals in tertiles 2 and 3 with events rates of 28% (HR:0.72; 95% CI: 0.46–1.12) and 21% (HR 0.79; 95% CI: 0.5–1.25) respectively.. Relative to the lowest U_Na_ tertile, those in tertile 2 and 3 were 39% (HR: 0.61; 95% CIs: 0.41, 0.91) and 10% (HR: 0.90; 95% CIs: 0.62, 1.33) less likely to experience to experience all-cause mortality. The multivariable Cox model had acceptable prediction precision (Harrell's C concordance measure 0.72).

**Conclusion:**

U_Na_ was a significant predictor of all-cause mortality but not MACE outcomes over 28–33 years with 173–229 mmol/day appearing to be the optimal level. U_Na_ may represent an emerging long-term prognostic biomarker that warrants further investigation.

**Supplementary Information:**

The online version contains supplementary material available at 10.1186/s12872-022-02830-3.

## Background

Heart failure (HF) is a clinical syndrome that occurs due an abnormality of cardiac structure or function. [1, 2]. It is estimated to occur in 1–2% of adults, with its prevalence increasing with age (≥ 10% in people > 70 years) [1]. Complications owing to HF incur significant morbidity, increased hospitalisations and associated length of stay [2].

There has been a growing interest in identifying biomarkers to enable prognostication of HF [3]. One such marker is urinary sodium. Due to the haemodynamic alterations in HF, reduced renal perfusion results in increased neurohormonal activation [4]. The renin-angiotensin system upregulation aims to increase fluid retention by conserving sodium and thereby reducing U_Na_ [5]. The reduced excretion of sodium and fluid therefore exacerbates a fluid overloaded state.

Many studies have demonstrated the association of low U_Na_ with poorer outcomes in acute HF patients including poor diuretic response during acute hospitalisation, increased risk of re-hospitalisation and an overall higher all-cause mortality [6–8]. However, there is limited evidence on the role of urinary sodium in predicting major adverse coronary events (MACE) in individuals with chronic HF over the long term. The objective of this study is to assess the long-term relationship between U_Na_ and MACE as well as all-cause mortality outcomes in men with HF over a period of 28–33 years.

## Methods

### Study participants data

**Fig. 1:**
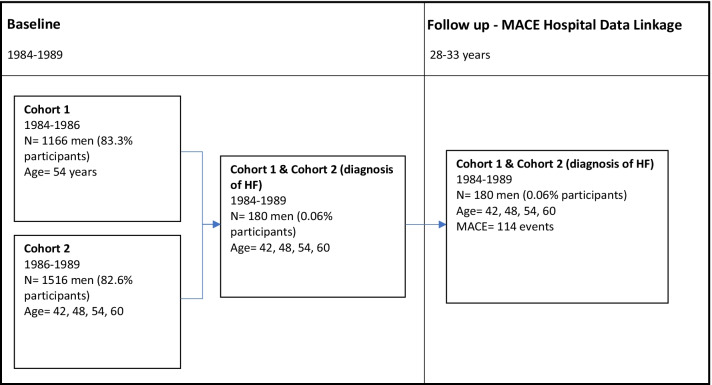
KIHD study design flowchart

Data for the current study included baseline, 24 h collection of urine which spanned 1984–1989, and incident MACE outcomes which were obtained through record linkage to national hospital discharge (Finnish Institute for Health and Welfare, Data License THL/93/5.05.00/2013) and death certificate databases (Statistics Finland, Data License TK-53-1770-16) 1984–2017. This is available from the corresponding author upon request. These data provided a follow-up period of 28–33 years. The baseline HF diagnosis refers to answering ‘yes’ to at least one of the following questions: ‘My doctor has told me that I have heart failure’ or ‘I have taken drugs for heart failure during the past seven days’. Participants were men from KIHD who had a self-reported HF diagnosis at baseline and provided 24-h urine samples (n = 180 of the n = 1956 enrolled) (Fig. 1).

### Study design

The Kuopio Ischaemic Heart Disease Risk Factor Study (KIHD) is an ongoing, prospective population-based cohort study investigating risk factors associated with CVD, atherosclerosis and related health conditions in Eastern Finnish men. A total of 2682 men who were 42, 48, 54, or 60 years old at baseline (83% of those eligible) were recruited in two cohorts between 1984 and 1989 (Fig. 1). The study design and recruitment details have been described elsewhere [9].The KIHD protocol was approved by the Research Ethics Committee of the University of Kuopio (December 1, 1983) and all participants provided informed consent.

### Variables of interest

#### Urinary sodium

The exposure variable was urinary sodium excretion (U_Na_), expressed as millimoles per day (mmol/day), which was categorized into tertiles. U_Na_ was calculated using sodium concentrations from a 24-h urine sample, collected in the 24 h prior to the study visit at baseline. This metric is the gold standard for measuring dietary sodium intake; collections accurately reflect sodium intake for 93% of the average global population [10]. In HF patients who are not taking loop diuretic medications, 24-h U_Na_ significantly correlates with dietary sodium estimates from food records of two consecutive days [11].

#### MACE outcomes and all-cause mortality

The primary outcome variable was time to major adverse coronary events (MACE) which included cardiovascular disease (CVD) death (excluding stroke death), acute myocardial infarction (AMI) death, and/or hospital presentation of AMI or unstable angina, monitored over a period of 28–33 years. Classification of suspected AMI events was coded according to the International Classification of Disease (ICD-10 codes I20 and I21-I22).

The secondary outcome was all-cause mortality which was based on register linkage to the causes of death register of Statistics Finland.

#### Covariates

Potential confounders were collected through self-reported questionnaires at baseline including dichotomized CVD family history, socio-economic status (SES), highest level of education (elementary school, elementary and vocational school, junior high, junior high and vocational school, or senior high), annual income, marital status, age, dichotomized smoker at baseline, alcohol intake (g/week), total physical activity (metabolic equivalents/hour/year), New York Heart Association classification, dichotomized currently taking medications including anti-hypertensives, beta-blockers, diuretics, insulin, and anti-hypercholesterolemic agents, diagnosis of diabetes (T1DM and T2DM combined into one variable as medical knowledge at baseline did not allow for distinguishing between the two) and hypertension, and a history of mental illness. Class of diuretics was also ascertained, based on their Anatomical Therapeutic Chemical code.

Body Mass Index (BMI), systolic (SBP) and diastolic blood pressure (DBP) were measured and blood collections were conducted between 8:00 and 10:00am after overnight fasting and both were conducted on the first baseline physical examination day by a trained nurse. Participants were also instructed to abstain from alcohol for three days and smoking for 12 h. Lipoprotein separation was conducted within three days of blood collection, and variables of interest include total cholesterol, low-density lipoproteins (LDL), and high-density lipoproteins (HDL).

### Statistical analyses

Study participants’ characteristics were summarized using mean (SD), median (IQR) and frequency (percent). Participant characteristics across tertiles of U_Na_ were compared using one-way ANOVA or Kruskal–Wallis-H test for continuous data and Chi-square test (or Fisher’s exact test) for categorical data. MACE rate across the study follow-up period (1984–2017) was investigated as the study primary outcome across tertiles of U_Na_ (primary exposure). Kaplan–Meier survival plots were used to explore MACE probability pattern across follow-up period at tertiles of U_Na_ and MACE rate per 1000 person years per annum and 95% confidence intervals (CI) at tertiles of U_Na_ were reported. In addition, a Cox proportional hazard regression model was used to estimate the age-adjusted hazard ratios (HRs) and their 95% confidence intervals (95% CIs) for tertiles of U_Na._ Potential confounders were investigated in trivariable Cox models (Additional file 1: Table S1) that included tertiles of U_Na_ and each potential confounder one at a time to identify important confounders. Goodness of fit measures (i.e. AIC, BIC, and Harrell's C concordance statistic) of the Cox model resulted from the backward stepwise variable selection method were compared with potential alternative models when additional predictors including CVD family history, socio-economic status (SES), dichotomized smoker at baseline, New York Heart Association classification, diagnosis of diabetes (T1DM and T2DM), dichotomized currently taking medications including anti-hypertensives, beta-blockers, and anti-hypercholesterolemic agents, and hypertension, and history of mental illness added into the multivariable Cox model (Additional file 2: Table S4) [12]. Inclusion of the above variables to the urinary tertiles did not improve the precision of model prediction nor altered HR estimations for tertiles of U_Na_. The proportional hazard assumptions were investigated graphically by log(-log(survival)) plots for U_Na_. Time to first MACE (survival) curves were illustrated using Kaplan–Meier estimator of the survival function using product limit estimator. Harrell’s C concordance statistic was reported aa a measure of model prediction precision. Values of Harrell’s C near 0.5 indicate that the risk score predictions are no better than a coin flip in determining which patient will live longer, and Harrell’s C values near 1 implies perfect concordance between risk score predictions and event times [13].

Urinary sodium was initially investigated as a continuous variable with no significant associations observed, therefore an exploratory analysis with tertiles was conducted. In additional sensitivity analysis, multivariable fractional polynomial (MFP) of continuous urinary sodium excretion was added into the model to assess non-linearity of urinary sodium as a continuous exposure in the multivariable Cox model (Additional file 2: Tables S1 and S2) [14]. Competing risk model of MACE versus non cardiovascular mortality as an alternative cause of failure on continuous urinary sodium excretion was performed as a sensitivity analysis.

Statistics were conducted using Stata version 17.0.

### Power calculation

A post-hoc power calculation was performed based on a total sample of 180 participants and an overall expected MACE rate of 60 per 1000 person years (extracted from the data). The study had 80% power at an alpha = 0.05 significance level to detect a minimum HR decline of 0.3 (HR = 0.7 or less) when comparing tertiles of U_Na_.

## Results

### Sample characteristics

Table 1 displays the sample baseline characteristics by exposure level (i.e. tertile). Participants were aged between 42 and 60 years (median = 54.4 years). Half of the sample were smokers at baseline, the majority did not complete junior high school, and most reported a family history of CVD. Diuretic use was greater in tertile 3 compared to tertiles 1 and 2, and hypertension more prevalent in tertile 1 compared to tertiles 2 and 3. Over half of the cohort were taking beta blocking agents, with the highest percentage found in tertile 3. Sixty six percent and 15% had self-reported hypertension and diabetes, respectively, and 14% of participants reported both hypertension and diabetes. Details of medication class and type as well as other characteristics are displayed in Table 1.

**Table 1:** Key baseline characteristics of sample

	Tertile 1 (< 173 mmol/day) (n = 61)	Tertile 2 (173–229 mmol/day) (n = 59)	Tertile 3 (230–491 mmol/day) (n = 60)	All Participants (n = 180)
Age, years, median (IQR)	54.42 (54.33, 55.00)	54.42 (54.33, 60.08)	54.42 (54.25, 54.50)	54.42 (54.33, 54.75)
Smoker, n (%)	20 (32.79)	13 (22.03)	15 (25.00)	48 (26.67)
Education level, n (%)				
Elementary school with vocational school or below	58 (95.08)	54 (91.52)	56 (93.33)	168 (93.33)
Junior high and above	3 (4.92)	5 (8.47)	4 (6.67)	12 (6.67)
Annual income Euro, median, (IQR)	8,074 (4,878, 13,036)	7,569 (5,382, 12,111)	7,401 (4,962, 11,606)	7,569 (5,046, 12,111)
Marital status, married, n (%)	51 (83.61)	52 (88.14)	51 (85.00)	154 (85.56)
BMI, median, (IQR)	27.16 (26.01, 30.74)	27.53 (25.83, 29.25)	29.95 (27.04, 31.88)	28.17 (26.09, 30.63)
Family history of CVD, n (%)	55 (90.16)	54 (91.53)	57 (95.00)	166 (92.22)
Currently taking medications, n (%)				
Hypertensives	45 (73.77)	40 (67.80)	41 (68.33)	126 (70.00)
Diuretics	19 (31.15)	17 (28.81)	26 (43.33)	62 (34.44)
High cholesterol	1 (1.64)	1 (1.69)	1 (1.67)	3 (1.67)
Beta blocking agents	39 (64.00)	37 (62.71)	31 (51.67)	107 (59.44)
Diuretics class, n (%)				
Hydrochlorothiazide and potassium-sparing agents	1 (1.64) *	5 (8.47) *	2 (3.33) *	8 (4.44)*
Hydrochlorothiazide	–	–	3 (5.00)*	3 (1.67)*
Furosemide and potassium-sparing agents	1 (1.64)*	–	2 (3.33)*	3 (1.67)*
Furosemide	1 (1.64)*	1 (1.69)*	3 (5.00)*	5 (2.78)*
Triamterene	–	1 (1.69)*	–	1 (0.56)*
Medical conditions, n (%)				
Diabetes (T1DM and T2DM)	9 (14.75)	7 (11.86)	11 (18.33)	27 (15.00)
Hypertension	45 (75.00)	38 (65.52)	33 (56.90)	116 (65.91)

*BMI* body mass index, *CVD* cardiovascular disease, *T1DM* type-1 diabetes mellitus, *T2DM* type-2 diabetes mellitus

*42 missing values

One hundred and fourteen MACE events were recorded over median 10 (IQR 3, 17) years with n = 42, 37, and 35 MACE events across tertiles 1, 2, and 3 respectively (Table 2). The rate of event per 1000 person years was 55.89, 37.85, and 40.77, across tertiles 1, 2, and 3 respectively, with the lowest MACE events observed in tertile 2. The median follow time was across tertiles 1, 2 and 3 were 10.3 years, 14.3 years, and 12.8 years respectively.

**Table 2:** MACE rates (n/1000), and unadjusted HR for tertiles of urinary sodium excretion with their 95% confidence interval

	Tertile 1 (< 173 mmol/day) (n = 61)	Tertile 2 (173 -229 mmol/day) (n = 59)	Tertile 3 (230–491 mmol/day) (n = 60)
Median survival analysis time (years) [IQR]	10.3 years [3.6–20.9]	14.3 years [7.5–27.2]	12.8 years [12.8–21.4]
MACE	42	37	35
total follow-up (1000 person years)	0.75	0.97	0.86
Rate (per 1000 person years)^2^	55.89 (41.30, 75.62)	37.85 (27.42, 52.23)	40.77 (29.28, 56.79)
Unadjusted HR	REF	0.68 (0.43, 1.05)	0.73 (0.46, 1.14)
*P* value	REF	0.084	0.163

### Urinary sodium excretion and MACE outcomes

In unadjusted models, relative to tertile 1, those in tertile 2 and 3 were 32% (HR 0.68; 95% CIs 0.43, 1.05) and 27% less likely to have a MACE event (HR 0.73; 95% CIs 0.46, 1.14), respectively however neither association was statistically significant (Table 2).

Table 3 shows the magnitude of these associations after adjustment for covariates. The trend between tertile 1 and 2 observed in the unadjusted model was lost when adjusted for age and full adjustment for age, smoking, beta blocking agents, diabetes, and mean diastolic blood pressure.

**Table 3:** Cox survival analysis: Hazard ratio (HR) and 95% confidence interval of MACE rate across tertiles of urinary sodium excretion

Urinary sodium excretion	HR	95%CI	Chi^2*^	*p* value
Model 1			3.43	0.18
Tertile 1	1.00	Reference		
Tertile 2	0.68	0.44, 1.05		
Tertile 3	0.73	0.46, 1.14		
Model 2^#^			3.03	0.22
Tertile 1	1.00	Reference		
Tertile 2	0.69	0.44, 1.07		
Tertile 3	0.76	0.48, 1.19		
Model 3^^^			2.25	0.32
Tertile 1	1.00	Reference		
Tertile 2	0.72	0.46, 1.12		
Tertile 3	0.79	0.50, 1.25		

^#^Model adjusted for age, ^^^model adjusted for age, smoking, beta-blocking agents, mean diastolic blood pressure, and diabetes, tertile 1 < 173 mmol/day, tertile 2 = 173–229 mmol/day, tertile 3 = 230–491 mmol/day, * d.f. = 2, Chi^2^ results are from testing of Cox regression beta coefficients

**Fig. 2:**
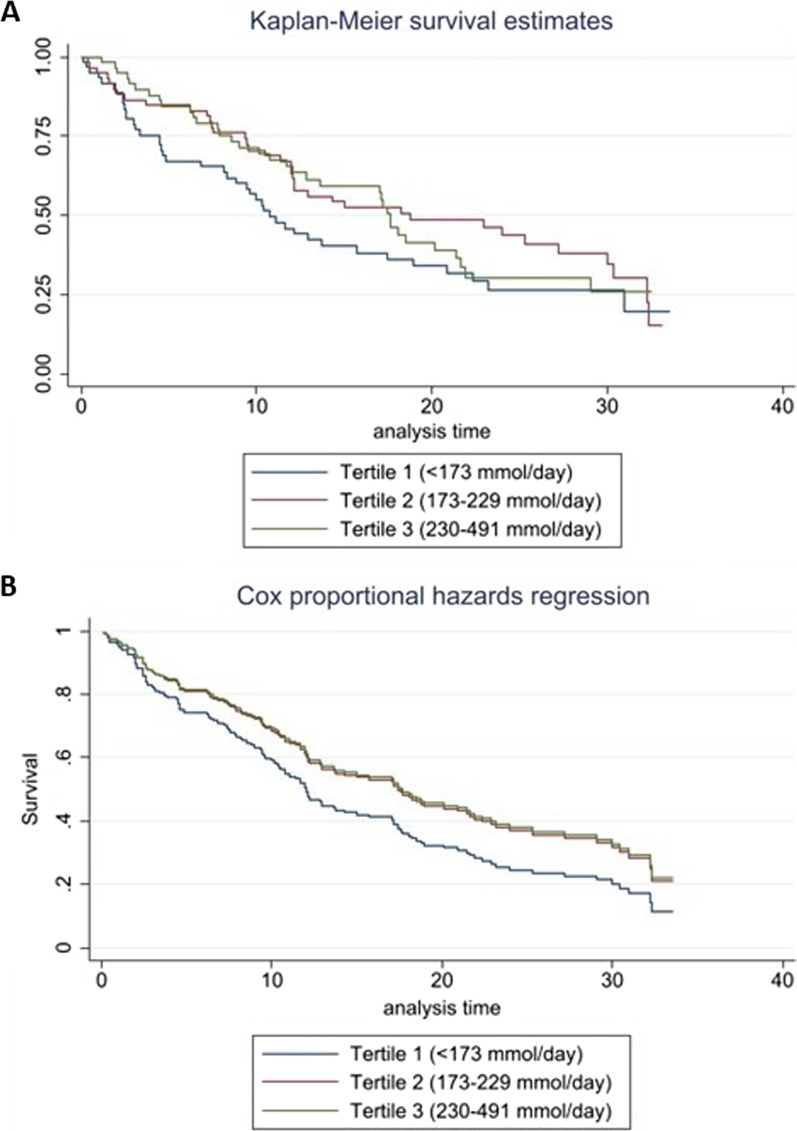
**A** Kaplan–Meier survival estimates of tertiles of urinary sodium for years to MACE, **B** Cox proportional hazards regression of tertiles of urinary sodium for years to MACE

Kaplan–Meier survival estimates displayed in Fig. 2A demonstrated the extent to which U_Na_ groups diverged over the analysis period with respect to time to MACE. Tertile 2 in comparison to Tertile 1 was non-significant with time to MACE events at commencement and a subsequent convergence after year 30 of follow up. Tertile 3 compared to tertile 1 until year 22 was noted to have a higher survival rate, following which, the survival time converged for tertiles 1 and 3. Figure 2B provides model-adjusted time to MACE for the final model.

### Urinary sodium excretion and all-cause mortality

One hundred and fifty all-cause deaths were recorded over median 19 (IQR 9, 28) years with n = 54, 46, and 50 MACE events across tertiles 1, 2, and 3 respectively (Table 4). The rate of event per 1000 person years was 54.16, 36.29, and 48.10, across tertiles 1, 2, and 3 respectively, with the lowest MACE events observed in tertile 2. In unadjusted models, relative to tertile 1, those in tertile 2 were 39% less likely to experience all-cause mortality over the follow up period (HR: 0.61; 95% CIs: 0.41, 0.91), and significance remained following adjustment for beta blocking agents, income, age, smoking, BMI, and diabetes. The multivariable Cox model had acceptable prediction precision as measured by Harrell’s C concordance statistic (Harrell’s C = 0.72). Those in tertile 3 were 10% less likely to experience all-cause mortality (HR 0.90; 95% CIs 0.62, 1.33), although this comparison was not significant (Table 5).

**Table 4:** All-cause mortality rates (n/1000), and unadjusted HR for tertiles of urinary sodium excretion with their 95% confidence interval

	Tertile 1 (< 173 mmol/day) (n = 61)	Tertile 2 (173–229 mmol/day) (n = 59)	Tertile 3 (230–491 mmol/day) (n = 60)
Median survival analysis time (years) [IQR]	10.3 years [3.6–20.9]	14.3 years [7.5–27.2]	12.8 years [12.8–21.4]
All-cause mortality	54	46	50
total follow-up (1000 person years)	1.00	1.27	1.04
Rate (per 1000 person years)^2^	54.16 (41.48, 70.71)	36.29 (27.18, 48.45)	48.10 (36.45, 63.46)
Unadjusted HR	REF	0.61 (0.41, 0.91)	0.90 (0.62, 1.33)
*P* value	REF	0.014	0.609

**Table 5:** Cox survival analysis: Hazard ratio (HR) and 95% confidence interval of all-cause mortality rate across tertiles of urinary sodium excretion

Urinary sodium excretion	HR	95%CI	Chi^2*^	*p* value
Model 1			6.51	0.04
Tertile 1	1.00	Reference		
Tertile 2	0.61	0.41, 0.91		
Tertile 3	0.90	0.62, 1.33		
Model 2^#^			6.57	0.04
Tertile 1	1.00	Reference		
Tertile 2	0.62	0.42, 0.92		
Tertile 3	0.96	0.65, 1.42		
Model 3^^^			6.57	0.04
Tertile 1	1.00	Reference		
Tertile 2	0.61	0.40, 0.91		
Tertile 3	0.93	0.61, 1.42		

^#^Model adjusted for age, ^^^model adjusted for beta blocking agents, income, age, smoking, BMI, and diabetes, tertile 1 < 173 mmol/day, tertile 2 = 173–229 mmol/day, tertile 3 = 230–491 mmol/day, * d.f. = 2, Chi^2^ results are from testing of Cox regression beta coefficients

MFP was used to investigate curvature in urinary sodium when included as a continuous exposure in the multivariable Cox model (Additional file 2: Tables S1 and S2). While the model showed an improvement in model goodness of fit the fractional polynomial term was not statistically significant (*p*-value = 0.185).

## Discussion

This study is the first of its kind to use long-term, prospective data to assess the relationship between U_Na_ and MACE as well as all-cause mortality outcomes in men with HF. Our data indicates U_Na_ is may be of prognostic value regarding all-cause mortality but less so for MACE. This association appeared to be U-shaped in nature, which has been previously seen U_Na_ and MACE events [15] as well as for incident heart failure [16].

An explanation for a lack of association between U_Na_ and MACE outcomes in this study could be due to type 1 error owing to a lack of statistical power due to the limited number of events in the KIHD cohort. A significant association was observed between U_Na_ and all-cause mortality events for which there were a greater number of events. Indeed, those with U_Na of_ 173–229 mmol/day had the lowest likelihood of all-cause mortality suggesting thereby suggesting there may be an optimal U_Na_ target for the purpose of self-management. Our study, provides a rationale for further investigation of its prognostic accuracy in a larger, more inclusive sample.

The potential prognostic value of U_Na_ is corroborated by the literature from acute decompensated HF. Low U_Na_ has a strong correlation with poor prognosis for hospitalised patients and increased length of stay [7, 17]. It is thought to be associated with HF due to reduced ejection fraction resulting in compensatory upregulation of the renin angiotensin system [18]. Furthermore, poor response to diuretic therapy in patients with HF is associated with a worse prognosis [16]. On the other hand, high U_Na_ is a reflection of dietary sodium intake, which correlates with a greater risk of developing HF [19]. Recommendations from current guidelines globally on daily salt intake in HF patients vary significantly, potentially due to lack of conclusive evidence [20–22]. However, emerging literature reveal a paradoxical association between low dietary sodium and a worse HF prognosis [23]. Due to the ambiguity of the role of dietary sodium, a large multi-centre randomised control trial (SODIUM-HF) is currently in progress aiming to elucidate the effect of dietary sodium < 1500 mg per day compared to current HF guideline dietary recommendations [24].

While there was a general U shaped trend noted in our study, this finding is not widely reflected in the literature. Martens and colleagues noted an inverse relationship between urinary sodium and risk of acute decompensation in patients with stable HF at baseline [25]. This variation in results could be due to the outcome measure; our study assessed MACE, in lieu of acute decompensation events. Furthermore, as the participants enrolled had a prior diagnosis of HF, they may have received dietary education at the time of initial diagnosis.

Another explanation could be dosing and compliance with HF pharmacotherapy which was not assessed. Diuretics, regardless of their class, exert their effect by increasing excretion of sodium and therefore water [26]. Dose reduction, possibly as a consequence of haemodynamic instability or acute biochemical marker derangements in addition to non-adherence with this class of medication can give rise to skewed results. In the KIHD cohort, 34.44% of participants at baseline were prescribed diuretics, however, with the limited data available summarising diuretics class (Table 1), no conclusions can be drawn regarding the effects of individual classes on MACE outcomes. Whilst evidence indicates that poor-response to diuretic use is associated with a worse HF prognosis, there are limited data on mortality benefit with certain types of diuretics. More specifically, loop diuretics haven’t been shown to confer long-term prognostic benefit in HF [27]. As we were underpowered to conduct meaningful analyses by sub-class of diuretics and anti-hypertensive agents taken by participants, future research investigating the link between sodium and MACE outcomes in HF patients will likely benefit from a detailed assessment of both these classes of medications in order to tease out potential effects of individual classes.

All- cause mortality was also noted to have a U shape association with U_Na_ which is similar to the trend noted with MACE events. This could be a consequence of higher and lower U_Na_ having poor control of comorbidities such as respiratory diseases, renal disease and malignancy which is known to contribute significantly toward non-cardiac mortality in patients with HF [28]. Due to the limited participant baseline screening data, it is difficult to elucidate the mechanism by which all-cause mortality is associated with U_Na_.

If these findings are replicated in a larger, more diverse sample, there may be implications for clinical practice with respect to the management of sodium intake in patients with HF. Evidence-based, best practice for managing HF with reduced ejection fraction (HFrEF) includes pharmacotherapy; angiotensin converting enzyme (ACE) inhibitors/ angiotensin receptor blocker (ARB), beta-blockers, mineralocorticoid receptor antagonists (MRAs), angiotensin receptor-neprilysin inhibitor (ARNI), hydralazine, nitrates and omega 3 polyunsaturated fatty acids. More recent evidence also supports the beneficial role of sodium glucose co-transporter type 2 (SGLT-2) inhibitors in reducing cardiovascular death rate and HF associated hospitalisation [29, 30]. While this therapeutic approach can help manage symptoms, prolong survival and improve quality of life (QoL) [21], morbidity and mortality remains high despite optimal therapy [31]. The PARADIGM-HF study showed that CHF patients receiving optimal therapy still had a 23% chance of cardiovascular death or CHF hospitalisation over the next 27 months [32]. In contrast, optimal management for heart failure with preserved ejection fraction (HFpEF) is not well understood. With the exception of MRAs and more recently SGLT-2 inhibitors, there is no clear evidence to support use of ACE/ARB, beta blockers, ARNI, calcium channel blockers and nitrates [33, 34]. This could be attributed to the various causes of HFpEF; the lack of etiological homogeneity preventing a clear consensus on prognostically beneficial pharmacotherapy. In light of this and our findings, dietary interventions may provide a safe, attractive approach to self-management of HF while encouraging adequate nutrition in patients whose condition can cause nutrient deficiencies and would benefit from further investigation. There is, however, a dearth of efficacy or acceptability data on dietary interventions in HF populations. US, Australian and European guidelines highlighted significant gaps in this area [21] and there remain no evidence-based recommendations beyond expert opinion.

### Limitations

This study focuses on an area of HF management with a relatively limited evidence base. It provides long-term longitudinal data pertaining to U_Na_ in HF patients, measured using gold standard 24 h urine collection. However, the relatively small sample size may have contributed to the association between U_Na_ and MACE being attenuated. Secondly, the external validity of this study may be limited by the homogeneity of the participants, given recruitment was limited to Eastern Finnish men and therefore these findings cannot be generalized to females. Furthermore, participant’s dietary sodium intake was not measured. Diuretic agents were also not withheld during baseline and subsequent U_Na_ measurement which may have resulted a left skew in the measured U_Na_. The MACE outcomes could not be stratified based on current HF directed therapy and anti-hyperglycaemic agents owing to limited data availably pertaining to the class of diuretics and anti-hypertensive agents in the KIHD cohort. Due to the variation in the median follow up across the urinary tertile groups, there may exist time in survival analysis bias. Lastly, as the urinary sodium was held constant for analysis of confounders, they may be factors dependent on the real time variability of urinary sodium that may not have been accounted.

## Conclusion

The current study suggests that further investigation of the long-term, prognostic value of 24 h U_Na_ may be warranted in HF patients. Future research in this area would benefit from the inclusion of detailed medication classes and the inclusion of a large number of patients, representative of the wider patient population, including women and other ethnic groups, with characterisation of HF sub-type.

## Supplementary Information

**Additional file 1: Sup. Table 1:** Potential confounders identified from trivariable Cox models for MACE events. **Sup. Table 2:** Inclusion of Model 4 representing a priori confounders. **Sup. Figure 1:** Proportional hazard assumption graph.

**Additional file 2: Sup. Table 1:** Multivariable fractional polynomial of MACE rate on continuous urinary sodium excretion. **Sup. Table 2:** Comparison of ordinary Cox model and MFP Cox model of MACE rate on continuous urinary sodium excretion. **Figure S1:** Observed versus model-based survival curves: left) fractional-polynomial multivariable Cox model; right) multivariable Cox model. **Sup. Table 3:** Competing risk model of MACE rate on continuous urinary sodium excretion with death by non-CV causes as alternative cause of failure. **Sup. Table 4:** Testing for potential confounders and goodness of fit comparison for alternative models. **Sup. Table 5:** Model prediction with and without inclusion of urinary tertiles

## Data Availability

Other relevant data can be found in the Supplementary Material. Additional data is available from the corresponding author upon request.

## References

[1] Ponikowski P, Voors AA, Anker SD, Bueno H, Cleland JG, Coats AJ, Falk V, González-Juanatey JR, Harjola VP, Jankowska EA (2016). 2016 ESC Guidelines for the diagnosis and treatment of acute and chronic heart failure: the task force for the diagnosis and treatment of acute and chronic heart failure of the European Society of Cardiology (ESC). Developed with the special contribution of the Heart Failure Association (HFA) of the ESC. European J Heart Fail.

[2] Chamberlain AM, Dunlay SM, Gerber Y, Manemann SM, Jiang R, Weston SA, Roger VL (2017). Burden and timing of hospitalizations in heart failure: a community study. Mayo Clin Proc.

[3] Ibrahim NE, Januzzi JL (2018). Established and emerging roles of biomarkers in heart failure. Circ Res.

[4] Orsborne C, Chaggar PS, Shaw SM, Williams SG (2017). The renin-angiotensin-aldosterone system in heart failure for the non-specialist: the past, the present and the future. Postgrad Med J.

[5] Bansal S, Lindenfeld J, Schrier RW (2009). Sodium retention in heart failure and cirrhosis: potential role of natriuretic doses of mineralocorticoid antagonist?. Circ Heart Fail.

[6] Damman K, Ter Maaten JM, Coster JE, Krikken JA, van Deursen VM, Krijnen HK, Hofman M, Nieuwland W, van Veldhuisen DJ, Voors AA, van der Meer P (2020). Clinical importance of urinary sodium excretion in acute heart failure. European J Heart Fail.

[7] Cunningham JW, Sun J-L, Mc Causland FR, Ly S, Anstrom KJ, Lindenfeld J, Givertz MM, Stevenson LW, Lakdawala NK (2020). Lower urine sodium predicts longer length of stay in acute heart failure patients: Insights from the ROSE AHF trial. Clin Cardiol.

[8] Hodson DZ, Griffin M, Mahoney D, Raghavendra P, Ahmad T, Turner J, Wilson FP, Tang WHW, Rao VS, Collins SP, Mullens W, Testani JM (2019). Natriuretic Response Is Highly Variable and Associated With 6-Month Survival: Insights From the ROSE-AHF Trial. JACC Heart Fail.

[9] Salonen J (1988). Is there a continuing need for longitudinal epidemiologic research? the Kuopio Ischaemic Heart Disease Risk Factor Study. Ann Clin Res.

[10] Lucko AM, Doktorchik C, Woodward M, Cogswell M, Neal B, Rabi D, Anderson C, He FJ, MacGregor GA, L'Abbe M (2018). Percentage of ingested sodium excreted in 24-hour urine collections: a systematic review and meta-analysis. J Clin Hypertens.

[11] Arcand J, Floras JS, Azevedo E, Mak S, Newton GE, Allard JP (2011). Evaluation of 2 methods for sodium intake assessment in cardiac patients with and without heart failure: the confounding effect of loop diuretics. Am J Clin Nutr.

[12] Kleinbaum DG, Klein M. Logistic regression: a self-learning text: Springer New York; 2010.

[13] Longato E, Vettoretti M, Di Camillo B (2020). A practical perspective on the concordance index for the evaluation and selection of prognostic time-to-event models. J Biomed Inform.

[14] Sauerbrei W, Royston P, Look M (2007). A new proposal for multivariable modelling of time-varying effects in survival data based on fractional polynomial time-transformation. Biom J.

[15] O’Donnell M, Mente A, Rangarajan S, McQueen MJ, O’Leary N, Yin L, Liu X, Swaminathan S, Khatib R, Rosengren A. Joint association of urinary sodium and potassium excretion with cardiovascular events and mortality: prospective cohort study. bmj. 2019;364. 10.1136/bmj.l772 PMC6415648 30867146

[16] Pfister R, Michels G, Sharp SJ, Luben R, Wareham NJ, Khaw KT (2014). Estimated urinary sodium excretion and risk of heart failure in men and women in the EPIC-Norfolk study. Eur J Heart Fail.

[17] Ferreira JP, Girerd N, Medeiros PB, Santos M, Carvalho HC, Bettencourt P, Kénizou D, Butler J, Zannad F, Rossignol P (2016). Spot urine sodium excretion as prognostic marker in acutely decompensated heart failure: the spironolactone effect. Clin Res Cardiol.

[18] Zucker IH, Xiao L, Haack KKV (2014). The central renin-angiotensin system and sympathetic nerve activity in chronic heart failure. Clin Sci.

[19] Kagiyama S, Koga T, Kaseda S, Ishihara S, Kawazoe N, Sadoshima S, Matsumura K, Takata Y, Tsuchihashi T, Iida M (2009). Correlation between increased urinary sodium excretion and decreased left ventricular diastolic function in patients with type 2 diabetes mellitus. Clin Cardiol Int Index Peer-Rev J Adv Treat Cardiovasc Dis.

[20] Hummel SL, Konerman MC (2016). Dietary sodium restriction in heart failure: a recommendation worth its salt?. JACC Heart Fail.

[21] Atherton JJ, Sindone A, De Pasquale CG, Driscoll A, MacDonald PS, Hopper I, Kistler PM, Briffa T, Wong J, Abhayaratna W (2018). National heart foundation of Australia and cardiac society of Australia and New Zealand: guidelines for the prevention, detection, and management of heart failure in Australia 2018. Heart Lung Circ.

[22] Gupta D, Georgiopoulou VV, Kalogeropoulos AP, Dunbar SB, Reilly CM, Sands JM, Fonarow GC, Jessup M, Gheorghiade M, Yancy C (2012). Dietary sodium intake in heart failure. Circulation.

[23] Khan MS, Jones DW, Butler J (2020). Salt, no salt, or less salt for patients with heart failure?. Am J Med.

[24] Colin-Ramirez E, Ezekowitz JA (2018). Rationale and design of the study of dietary intervention under 100 MMOL in heart failure (SODIUM-HF). Am Heart J.

[25] Martens P, Dupont M, Verbrugge FH, Damman K, Degryse N, Nijst P, Reynders C, Penders J, Tang WW, Testani J (2019). Urinary sodium profiling in chronic heart failure to detect development of acute decompensated heart failure. JACC Heart Fail.

[26] Casu G, Merella P (2015). Diuretic Therapy in heart failure–current approaches. European Cardiology Review.

[27] Kapelios CJ, Malliaras K, Kaldara E, Vakrou S, Nanas JN (2018). Loop diuretics for chronic heart failure: a foe in disguise of a friend?. European Heart J Cardiovasc Pharmacother.

[28] Vergaro G, Ghionzoli N, Innocenti L, Taddei C, Giannoni A, Valleggi A, Borrelli C, Senni M, Passino C, Emdin M (2019). Noncardiac versus cardiac mortality in heart failure with preserved, midrange, and reduced ejection fraction. J Am Heart Assoc.

[29] Zannad F, Ferreira JP, Pocock SJ, Anker SD, Butler J, Filippatos G, Brueckmann M, Ofstad AP, Pfarr E, Jamal W (2020). SGLT2 inhibitors in patients with heart failure with reduced ejection fraction: a meta-analysis of the EMPEROR-reduced and DAPA-HF trials. Lancet.

[30] Seferović PM, Coats AJ, Ponikowski P, Filippatos G, Huelsmann M, Jhund PS, Polovina MM, Komajda M, Seferović J, Sari I (2020). European society of cardiology/heart failure association position paper on the role and safety of new glucose-lowering drugs in patients with heart failure. Eur J Heart Fail.

[31] Ferrin PC, McCreath L, Navankasattusas S, Drakos SG (2016). Recovery versus remission: clinical insights. Heart Fail Clin.

[32] Okumura N, Jhund PS, Gong J, Lefkowitz MP, Rizkala AR, Rouleau JL, Shi VC, Swedberg K, Zile MR, Solomon SD (2016). Effects of sacubitril/valsartan in the PARADIGM-HF trial (prospective comparison of ARNI with ACEI to determine impact on global mortality and morbidity in heart failure) according to background therapy. Circ Heart Fail.

[33] Henning RJ (2020). Diagnosis and treatment of heart failure with preserved left ventricular ejection fraction. World J Cardiol.

[34] Anker SD, Butler J, Filippatos G, Ferreira JP, Bocchi E, Böhm M, Brunner–La Rocca HP, Choi DJ, Chopra V, Chuquiure-Valenzuela E (2021). Empagliflozin in heart failure with a preserved ejection fraction. N Engl J Med.

