# Supplementary material for: Urinary sodium concentration predicts time to major adverse coronary events and all-cause mortality in men with heart failure over a 28–33-year period: a prospective cohort study
Source: BMC Cardiovasc Disord. 2022 Sep 2;22:391. doi: 10.1186/s12872-022-02830-3 (PMC9438140; doi:10.1186/s12872-022-02830-3)
Supplement: Supplementary file 1 — Additional file 1: Sup. Table 1: Potential confounders identified from trivariable Cox models for MACE events. Sup. Table 2: Inclusion of Model 4 representing a priori confounders. Sup. Figure 1: Proportional hazard assumption graph. [file 12872_2022_2830_MOESM1_ESM.pdf]

ADDITIONAL FILE 1

(Sup. Table 1) Potential confounders identified from trivariable Cox models for MACE events

	HR	95%CI	p value
**Age**	1.07	1.00, 1.13	0.04
U_{Na} tertile 1	Ref		
U_{Na} tertile 2	0.69	0.44, 1.07	0.09
U_{Na} tertile 3	0.76	0.48, 1.1932	0.22
**Annual income**	0.99	0.99, 0.99	0.03
U_{Na} tertile 1	Ref		
U_{Na} tertile 2	0.64	0.41, 1.01	0.06
U_{Na} tertile 3	0.70	0.44, 1.10	0.12
**Smoking**	1.55	1.03, 2.33	0.04
U_{Na} tertile 1	Ref		
U_{Na} tertile 2	0.70	0.45, 1.09	0.12
U_{Na} tertile 3	0.74	0.47, 1.16	0.19
**Marital status**	1.36	1.05, 1.75	0.02
U_{Na} tertile 1	Ref		
U_{Na} tertile 2	0.67	0.43, 1.04	0.07
U_{Na} tertile 3	0.72	0.46, 1.13	0.15
**Diuretic drugs**	1.73	1.18, 2.54	0.01
U_{Na} tertile 1	Ref		
U_{na} tertile 2	0.63	0.40, 0.98	0.04
U_{Na} tertile 3	0.67	0.42, 1.05	0.08
**Beta-blocking agents**	1.93	1.31, 2.86	0.001

U_{Na} tertile 1	Ref		
U_{Na} tertile 2	0.68	0.43, 1.05,	0.08
U_{Na} tertile 3	0.79	0.50, 1.23	0.30
**Diabetes (T1DM and T2DM)**	2.01	1.28, 3.36	0.01
U_{Na} tertile 1	Ref		
U_{Na} tertile 2	0.67	0.43, 1.04	0.07
U_{Na} tertile 3	0.68	0.43, 1.07	0.10
**Hypertension**	2.02	1.33, 3.09	0.001
U_{Na} tertile 1	Ref		
U_{Na} tertile 2	0.72	0.46, 1.12	0.14
U_{Na} tertile 3	0.84	0.53, 1.32	0.45
**Mean diastolic blood pressure**	1.02	1.00, 1.04	0.02
U_{Na} tertile 1	Ref		
U_{Na} tertile 2	0.68	0.44, 1.06	0.09
U_{Na} tertile 3	0.71	0.45, 1.11	0.33
**Mean systolic blood pressure**	1.01	1.00, 1.02	0.02
U_{Na} tertile 1	Ref		
U_{Na} tertile 2	0.71	0.46, 1.11	0.14
U_{Na} tertile 3	0.69	0.44, 1.09	0.11

U_{Na} = Urinary sodium excretion, T1DM = type-1 diabetes mellitus, T2DM = type-2 diabetes mellitus, tertile 1 <173 mmol/day, tertile 2 = 173-229 mmol/day, tertile 3 = 230-491 mmol/day

Sup. Table 2) Inclusion of Model 4 representing *a priori* confounders

Urinary sodium excretion	HR	95%CI	Chi^{2} *	p value
Model 1			3.43	0.18
• Tertile 1	1.00	Reference		
• Tertile 2	0.68	0.44, 1.05		
• Tertile 3	0.73	0.46, 1.14		
Model 2#			3.03	0.22
• Tertile 1	1.00	Reference		
• Tertile 2	0.69	0.44, 1.07		
• Tertile 3	0.76	0.48, 1.19		
Model 3^			2.25	0.32
• Tertile 1	1.00	Reference		
• Tertile 2	0.72	0.46, 1.12		
• Tertile 3	0.79	0.50, 1.25		
Model 4^^			2.55	0.27
• Tertile 1	1.00			
• Tertile 2	0.65	0.43, 0.99		
• Tertile 3	0.80	0.53, 1.22		

#Model adjusted for age, ^model adjusted for age, smoking, beta-blocking agents, mean diastolic blood pressure, and diabetes, ^^model adjusted for diuretics, income, education, marital status, age, smoking, beta-blocking agents, mean diastolic blood

pressure, and diabetes, tertile 1 <173 mmol/day, tertile 2 = 173-229 mmol/day, tertile 3 = 230-491 mmol/day, * d.f. = 2, Chi^2 results are from testing of Cox regression beta coefficients

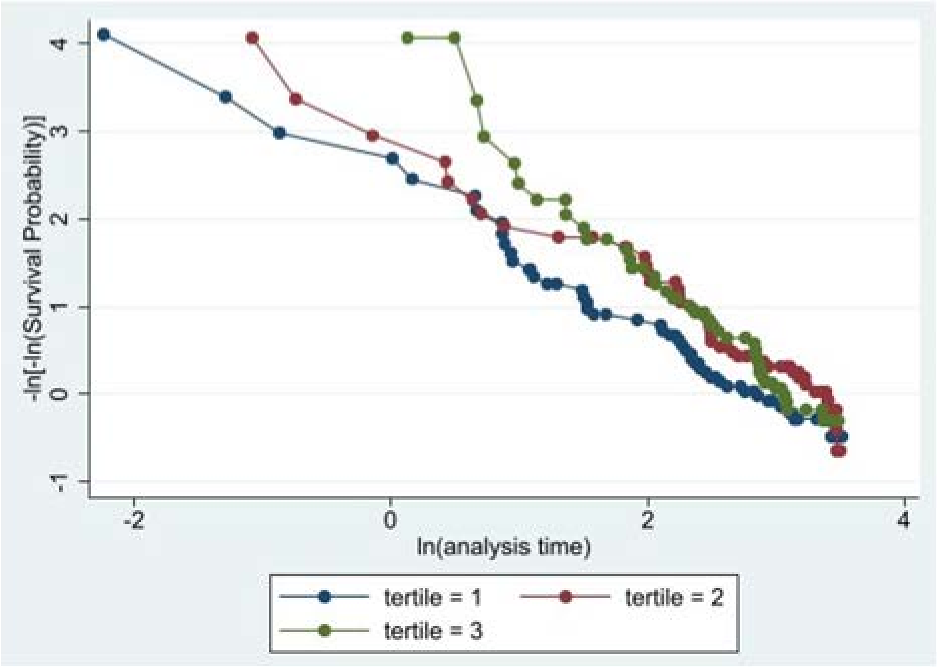

(Sup. Figure 1): Proportional Hazard assumption graph
