# Supplementary material for: Urinary sodium concentration predicts time to major adverse coronary events and all-cause mortality in men with heart failure over a 28–33-year period: a prospective cohort study
Source: BMC Cardiovasc Disord. 2022 Sep 2;22:391. doi: 10.1186/s12872-022-02830-3 (PMC9438140; doi:10.1186/s12872-022-02830-3)
Supplement: Supplementary file 2 — Additional file 2: Sup. Table 1: Multivariable fractional polynomial of MACE rate on continuous urinary sodium excretion. Sup. Table 2: Comparison of ordinary Cox model and MFP Cox model of MACE rate on continuous urinary sodium excretion. Figure S1: Observed versus model-based survival curves: left) fractional-polynomial multivariable Cox model; right) multivariable Cox model. Sup. Table 3: Competing risk model of MACE rate on continuous urinary sodium excretion with death by non-CV causes as alternative cause of failure. Sup. Table 4: Testing for potential confounders and goodness of fit comparison for alternative models. Sup. Table 5: Model prediction with and without inclusion of urinary tertiles [file 12872_2022_2830_MOESM2_ESM.pdf]

ADDITIONAL FILE 2

Multivariable fractional polynomial (MFP) approach was used to investigate potential non-linearity in urinary sodium as a continuous exposure in the multivariable Cox model.

Table 1 shows MFP model in a Cox regression that adjusts for age, education, income, smoking at baseline, diabetes, use of Diuretics and beta blocking agents. As Table 1 illustrates fractional polynomial was not statistically significant, however it improved model fit (Table 2) as measure with AIC, and BIC. However, due to complexity of interpreting non-linear effects of urinary sodium from MFP, exploratory data driven nature of the approach and (partially) non-significance of MFP terms we decided to present a model with tertile cut-off in the paper and present MFP in a supplement.

(Sup. Table 1) Multivariable fractional polynomial of MACE rate on continuous urinary sodium excretion

```
Cox regression with Breslow method for ties

No. of subjects =        176                            Number of obs =    744

No. of failures =        152

Time at risk    = 2,538.9897

--------------------------------------------------------------------------------

            _t | Coefficient  Std. err.      z    P>|z|     [95% conf. interval]

---------------+----------------------------------------------------------------

Education 2  |  -.1195795   .2414559    -0.50   0.620    -.5928244    .3536654

Education 3  |   .1364254   .2631054     0.52   0.604    -.3792517    .6521025
income       |  -5.98e-06   3.23e-06    -1.85   0.064    -.0000123    3.46e-07

          age |   .0907239   .0302643     3.00   0.003     .0314069    .1500408

      smoking |   .0184664   .0045554     4.05   0.000      .009538    .0273948

     diabetes |   .5767161   .2347294     2.46   0.014     .1166549    1.036777

          DBP |   .0107847   .0076772     1.40   0.160    -.0042623    .0258317

    Diuretics |   .3299891   .1916241     1.72   0.085    -.0455873    .7055654
Beta blocking|   .2969235    .192908     1.54   0.124    -.0811692    .6750161

       DU NA |    -.00341   .0018236                      -.0069842    .0001642

   DU NA  t1 |   .0022103   .0009236                        .0004       .0040206

   DU NA  t2 |  -.0020837   .0008581                      -.0037655   -.0004019
--------------------------------------------------------------------------------

Variable   Powers     P-value    Final

name       selected   FP2 v null deviance

----------------------------------------

duna        3 3        0.1852    1289.301

----------------------------------------
```

(Sup. Table 2) Comparison of ordinary Cox model and MFP Cox model of MACE rate on continuous urinary sodium excretion

Variable	Cox	Cox fractional polynomial
DU NA	-.00105795	-.00341
DU NA t1		.00221032
DU NA t2		-.00208367
education		
1	-.21335913	-.11957951
2	.23208104	.13642543
income	-9.051e-06	-5.984e-06
age	.13563662	.09072387
smoking	.02510053	.01846641
diabetes	.79336989	.57671613
DBP	.01860581	.01078469
Diuretics	.51190433	.32998905
Beta blocking	.20050481	.29692346
N	744	744
ll	-789.2262	-744.65034
chi^2	89.029573	56.500799
AIC	1598.4524	1413.3007
BIC	1644.5728	1468.6452
rank	10	12

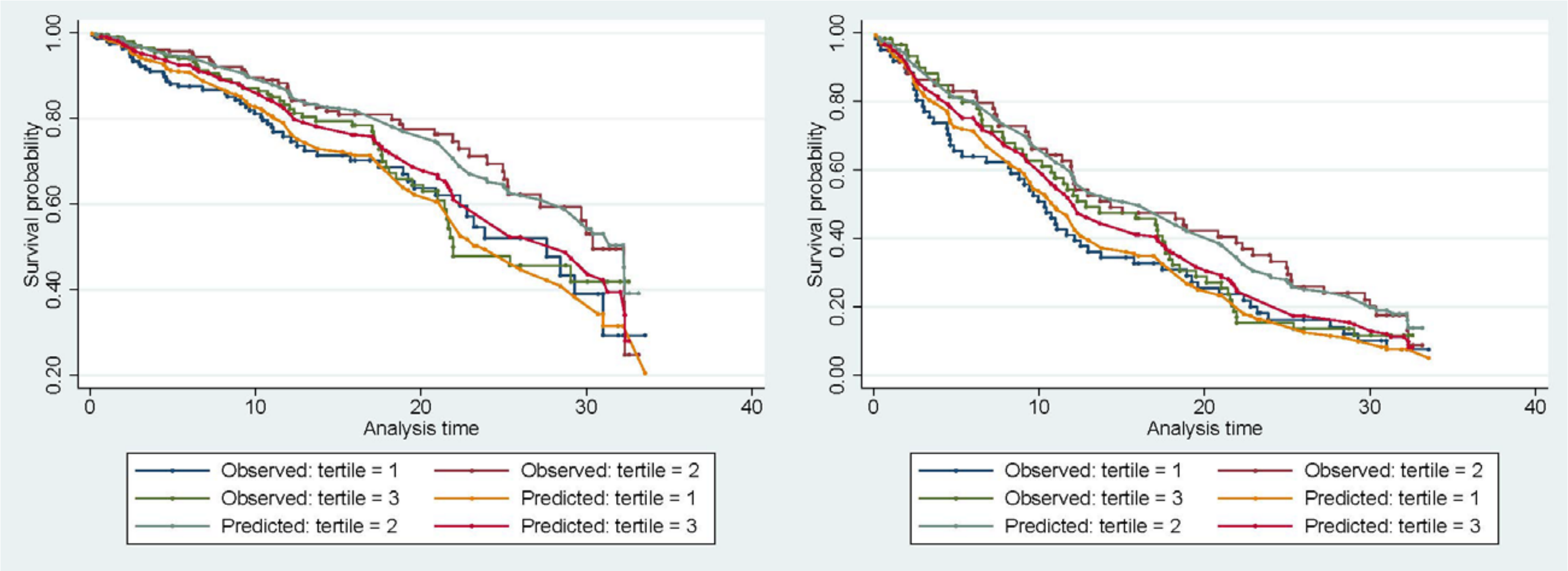

Figure S1. Observed versus model-based survival curves: left) fractional-polynomial multivariable Cox model; right) multivariable Cox model

A competing risk model of MACE rate on continuous urinary sodium excretion with death by non-CV causes as alternative type of failure was performed as a sensitivity analysis. The competing risk model results did not show a notable difference in hazard ratio estimation for continuous urinary sodium excretion when comparing the estimates with single failure Cox regression model.

(Sup. Table 3) Competing risk model of MACE rate on continuous urinary sodium excretion with death by non-CV causes as alternative cause of failure

_t	SHR	Robust std. err.	z	P>|z|	[95% conf.	interval]
DU NA	.9994835	.00161	-0.32	0.748	.996333	1.002644
education						
1	.7969757	.2782948	-0.65	0.516	.40199	1.580065
2	.9102766	.3373086	-0.25	0.800	.4403068	1.881877
income	.9999914	4.50e-06	-1.92	0.055	.9999826	1
age	1.043998	.0452406	0.99	0.320	.9589888	1.136542
smoking	.9967897	.005416	-0.59	0.554	.9862309	1.007462
diabetes	1.294004	.4224037	0.79	0.430	.6824592	2.453548
DBP	.999375	.0122821	-0.05	0.959	.9755901	1.02374
Diuretics	.9181688	.2479868	-0.32	0.752	.5407827	1.558914
Beta blocking	.8137418	.2107016	-0.80	0.426	.4898754	1.351723

Table 4 illustrated our model variable selection justification and compared goodness of fit (AIC, BIC, and Harrell's C) of potential alternative models when additional predictors including CVD family history, socio-economic status (SES), dichotomized smoker at baseline, New York Heart Association classification, diagnosis of diabetes (T1DM and T2DM), dichotomized currently taking medications including anti-hypertensives, beta-blockers, and anti-hypercholesterolemic agents, and hypertension, and history of mental illness were included in models. As shown in Table 4, no additional factor improved model prediction precision or resulted in better goodness of fit.

(Sup. Table 4) Testing for potential confounders and goodness of fit comparison for alternative models

Variable	Model1	Model2	Model3	Model4	Model5	Model6	Model7	Model8
DU NA tertile								
2	-.53867597	-.51477548	-.53113229	-.48873691	-.48253426	-.53896474	-.52097038	-.49112808
3	-.33988955	-.32532889	-.34594754	-.27798316	-.28931126	-.34351635	-.3425538	-.3386771
education								
1	-.10762518	-.05276402	-.09623985	-.11398755	-.01992576	-.11152876	-.14601342	-.14294279
2	.23963556	.62688278	.24628542	.24703215	.27693271	.23409295	.22931084	.22097902
age	.13916321	.15437671	.13923658	.13360692	.13567737	.13902846	.13443907	.12871022
smoking	.02353375	.0243801	.02371801	.02354826	.0263061	.02357489	.02421518	.02496953
diabetes	.80500601	.94685223	.8020295	.83068586	.84009129	.80297673	.79917097	.89241962
DBP	.01945778	.02015717	.01927564	.01551653	.0207273	.0196078	.02048309	.02326323
Diuretics	.56257267	.56743482	.56246222	.50499984	.35047661	.56017517	.52759568	.50961105
Beta blocking	.1902761	.25731878	.17318759	.11653974	-.41951852	.18619789	.23275967	.17190641

income	-9.478e-06		-9.226e-06	-9.261e-06	-8.946e-06	-9.503e-06	-.00001077	-.00001022
SES		.06269116						
NYHA			.04431328					
Hypertension				.22387962				
Drug HP					.885487			
CVD history						.04103463		
anti hypercholesterolemic							1.3457719	
History of mental illness								-.56793568
N	744	746	744	721	744	744	744	744
ll	-786.51672	-793.34333	-786.44332	-773.67517	-783.48669	-786.50846	-784.87797	-784.91962
chi^2	94.44853	93.517261	94.595345	87.460367	100.5086	94.465058	97.726033	97.642742
AIC	1595.0334	1608.6867	1596.8866	1571.3503	1590.9734	1597.0169	1593.7559	1593.8392
BIC	1645.7659	1659.4486	1652.2311	1626.318	1646.3179	1652.3614	1649.1004	1649.1837
Harrell's C	0.7257	0.7195	0.7259	0.7155	0.7327	0.7257	0.7276	0.7315
rank	11	11	12	12	12	12	12	12

(Sup. Table 5) Model prediction with and without inclusion of urinary tertiles

```
stcox i.tertile i.education v0227 ageyears packyear db4 diastka c03 c07,  base

No. of subjects =        176                            Number of obs =    176
No. of failures =        152
Time at risk    = 2,538.9897
                                                        LR chi2(11)   =  53.08
Log likelihood = -646.36315                             Prob > chi2   = 0.0000

------------------------------------------------------------------------------
          _t | Haz. ratio   Std. err.      z    P>|z|     [95% conf. interval]
-------------+----------------------------------------------------------------
     tertile |
          1  |          1  (base)
          2  |   .6891765   .1453199    -1.77   0.077     .4558752    1.041873
          3  |   .8307877   .1738239    -0.89   0.376     .5513089    1.251945
             |
   education |
          0  |          1  (base)
          1  |   .9685563   .2372356    -0.13   0.896     .5992856    1.565366
          2  |    1.18684   .3093839     0.66   0.511     .7120363    1.978255
             |
       v0227 |   .9999937   3.22e-06    -1.96   0.050     .9999874           1
    ageyears |   1.101384    .033539     3.17   0.002     1.037572     1.16912
    packyear |     1.0177   .0046726     3.82   0.000     1.008583    1.026899
         db4 |   1.828531   .4262646     2.59   0.010     1.157901    2.887575
     diastka |   1.011632   .0076986     1.52   0.129     .9966554    1.026834
         c03 |    1.44969   .2794426     1.93   0.054     .9935673    2.115207
         c07 |   1.312947   .2476653     1.44   0.149     .9071569    1.900256
------------------------------------------------------------------------------

. estimates store cox

. estimates table cox , stats(N ll chi2 aic bic rank)

-------------+------------
         aic |  1314.7263
         bic |  1349.6016
--------------------------

. estat concordance

        Failure _d: total_MACE_outcome
  Analysis time _t: MACE_time_from_baseline_ALL/365.25

Harrell's C concordance statistic

  Number of subjects (N)             =      176
  Number of comparison pairs (P)     =    14749
  Number of orderings as expected (E) =     9829
  Number of tied predictions (T)     =        0

          Harrell's C = (E + T/2) / P =   0.6664
                            Somers' D =   0.3328
```

```
. same mode without tertiles
. stcox i.education v0227 ageyears packyear db4 diastka c03 c07,  base


No. of subjects =         176                         Number of obs =    176
No. of failures =         152
Time at risk    = 2,538.9897
                                                      LR chi2(9)    =  49.95
Log likelihood = -647.92567                           Prob > chi2   = 0.0000

------------------------------------------------------------------------------
          _t | Haz. ratio   Std. err.      z    P>|z|     [95% conf. interval]
-------------+----------------------------------------------------------------
   education |
          0  |          1  (base)
          1  |   .9030482   .2168283    -0.42   0.671     .5640679    1.445741
          2  |   1.156028   .3007663     0.56   0.577     .6942393    1.924985
             |
       v0227 |   .9999942   3.16e-06    -1.84   0.066      .999988           1
    ageyears |   1.100111   .0330381     3.18   0.001     1.037226    1.166808
    packyear |   1.019059   .0045956     4.19   0.000     1.010092    1.028106
         db4 |   1.812965   .4196603     2.57   0.010     1.151743    2.853798
     diastka |   1.011534   .0077863     1.49   0.136     .9963879    1.026911
         c03 |   1.375654   .2583989     1.70   0.090     .9519668    1.987908
         c07 |   1.350842   .2490655     1.63   0.103     .9411567    1.938865
------------------------------------------------------------------------------

.
. estimates store cox

.
. estimates table cox , stats(N ll chi2 aic bic rank)

--------------------------
    Variable |    cox
-------------+------------
-------------+------------
         aic |  1313.8513
         bic |  1342.3857
--------------------------


.
. estat concordance

        Failure _d: total_MACE_outcome
  Analysis time _t: MACE_time_from_baseline_ALL/365.25

Harrell's C concordance statistic

  Number of subjects (N)             =      176
  Number of comparison pairs (P)     =    14749
  Number of orderings as expected (E) =     9746
  Number of tied predictions (T)     =        0

          Harrell's C = (E + T/2) / P =   0.6608
                             Somers' D =   0.3216
```
